# Cubic Hafnium Nitride: A Novel Topological Semimetal Hosting a 0-Dimensional (0-D) Nodal Point and a 1-D Topological Nodal Ring

**DOI:** 10.3389/fchem.2020.00727

**Published:** 2020-08-26

**Authors:** Yang Li, Jihong Xia

**Affiliations:** ^1^Department of Physics, Chongqing University of Arts and Sciences, Chongqing, China; ^2^Faculty of Mechanical and Electrical Engineering, Kunming University of Science and Technology, Kunming, China

**Keywords:** topological nodal ring, triple point, density functional theory, phonon dispersion, mechanical behaviors

## Abstract

Very recently, topological semimetals with nontrivial band crossing and associated topological surface states have received widespread attention. Various types of topological semimetals, including nodal point semimetals, nodal line semimetals, and nodal surface semimetals, have been predicted from first principles. In absence of spin-orbit coupling (SOC) effect, we propose that cubic-type hafnium nitride (HfN) with a $Pm\bar{3}m$ space group is a novel topological semimetal hosting a rare 0-D triple nodal point and a 1-D topological nodal ring. More importantly, the interesting 0-D and 1-D topological states all occur near the Fermi level, and these topological states are not disturbed by other extraneous bands. When the SOC effect is taken into consideration, 0-D triple nodal point was gapped and a new 0-D topological element, namely, Dirac point appears along Γ-R path. Finally, the dynamical and mechanical stabilities of this semimetal and its associated mechanical properties are discussed in order to provide a reference for future investigations. Our work promises that HfN can serve as a superior topological semimetal with high stability, excellent mechanical properties, and rich topological states.

## Introduction

After the discovery of topological insulators (Zhang et al., 2009; Hasan and Kane, 2010; Yu et al., 2010; Qi and Zhang, 2011; Rechtsman et al., 2013), topological semimetals (Jiang et al., 2015; Fang et al., 2016; Chang et al., 2017; Yan and Felser, 2017; Gao et al., 2019) with topological band inversion and exotic topological boundary states have attracted widespread attention. To date, many types of topological semimetals have been proposed, including topological nodal point semimetals (Liu et al., 2013, 2019; Li et al., 2014; Xu et al., 2014, 2016, 2020; Dvorak and Wu, 2015; Cheng et al., 2017; Zhong et al., 2017; Gao et al., 2019; Zhang et al., 2019), topological nodal line semimetals (Cai et al., 2018; Chen et al., 2018; Gao et al., 2018; Zhou et al., 2018; He et al., 2019; Jin et al., 2019a; Pham et al., 2019; Yi et al., 2019; Zou et al., 2019; Zhao et al., 2020), and topological nodal surface semimetals (Qie et al., 2019; Yang and Zhang, 2020).

For nodal point semimetals, the most studied members among them are Weyl and Dirac semimetals. Weyl semimetals (Hosur and Qi, 2013; Soluyanov et al., 2015; Zheng et al., 2016; Lin et al., 2017; Zhang M. et al., 2018; Bedoyapinto et al., 2020; Geishendorf et al., 2020; Thakur et al., 2020) possess twofold degenerate 0-D Weyl nodes, which are protected by inversion (P) or time-reversal (T) symmetries. However, Dirac semimetals (Chen et al., 2015; Gong et al., 2017; Yuan et al., 2017; Zhang X. et al., 2018; Jing and Heine, 2019) host quadruple-degenerate 0-D Dirac points, and these massless Dirac points are protected by the crystalline symmetry. In addition to the Weyl and Dirac semimetals generated by twofold- or quadruple-degenerate nodal points, topological nodal point semimetals with triple-degenerate 0-D nodal points have also become a new focus of research due to their novel topological states and related physics properties. Among inversion-asymmetric and centrosymmetric systems, some members are proposed to be triple point-type semimetals (He et al., 2017; Lv et al., 2017).

Moreover, topological nodal line semimetals (Cai et al., 2018; Chen et al., 2018; Zhou et al., 2018; Pham et al., 2019; Yi et al., 2019) possess 1-D topological elements, which are formed by band crossing along a line in momentum space. Compared to the topological nodal point semimetals with 0-D topological elements, topological nodal line semimetals feature more subtypes due to the fact that the 1-D line can deform into many different geometries. Topological nodal ring semimetals with a 1-D nodal loop in momentum space form one well-known type of topological nodal line semimetal. Topological nodal ring semimetals have emerged as a hot research topic very recently because these topological materials have intriguing electronic band behaviors and interesting drum-head-like surface states.

Topological materials are suitable for a range of potential applications, such as superconductivity (Das et al., 2012), chemical sensors (Schoop et al., 2018), and thermoelectricity (Sung et al., 2014). However, the candidates for such topological semimetals that possess more than one topological element are quite limited, which greatly restricts further investigations into those topological semimetals with rich topological elements. We would like to point out that materials with CsCl-type enjoy many interesting properties, such as simple in crystal structure, easy of synthesis and high phase stability, and thus, CsCl-type materials are a good target for searching for now functional materials. In this study, we propose, from first principles, that cubic (CsCl type) hafnium nitride (HfN), which is a phase-stable material, can serve as an interesting topological semimetal exhibiting both 0-D and 1-D topological elements. Furthermore, these topological elements all appear around the Fermi level. We hope the 0-D and 1-D topological elements, as well as the nontrivial surface states in cubic HfN, will soon be confirmed experimentally.

## Computational Details

All atomic and electronic structure calculations were performed by means of the Vienna *Ab initio* Simulation Package (VASP) (Sun et al., 2003) within the generalized gradient approximation (Perdew et al., 1996) using the Perdew–Burke–Ernzerhof (PBE) exchange-correlation functional. The projector augmented wave (PAW) (Perdew et al., 1998) pseudo-potential was employed with a cutoff energy of 600 eV for plane-wave expansions. The whole Brillouin zone is sampled with the Γ- centered *k* mesh of 11 × 11 × 11. The convergence criteria for energy and force were set at 10^−6^ eV per atom and 0.0005 eV/Å, respectively. The surface states of HfN were investigated in this study on a basis of a slab model built by WannierTools package (http://www.wanniertools.com) (Mostofi et al., 2008), according to the method of maximally localized Wannier functions. The value of NSlab, i.e., Number of slabs for slab band was set as 20 in this work.

## Results and Discussion

**Figure 1:**
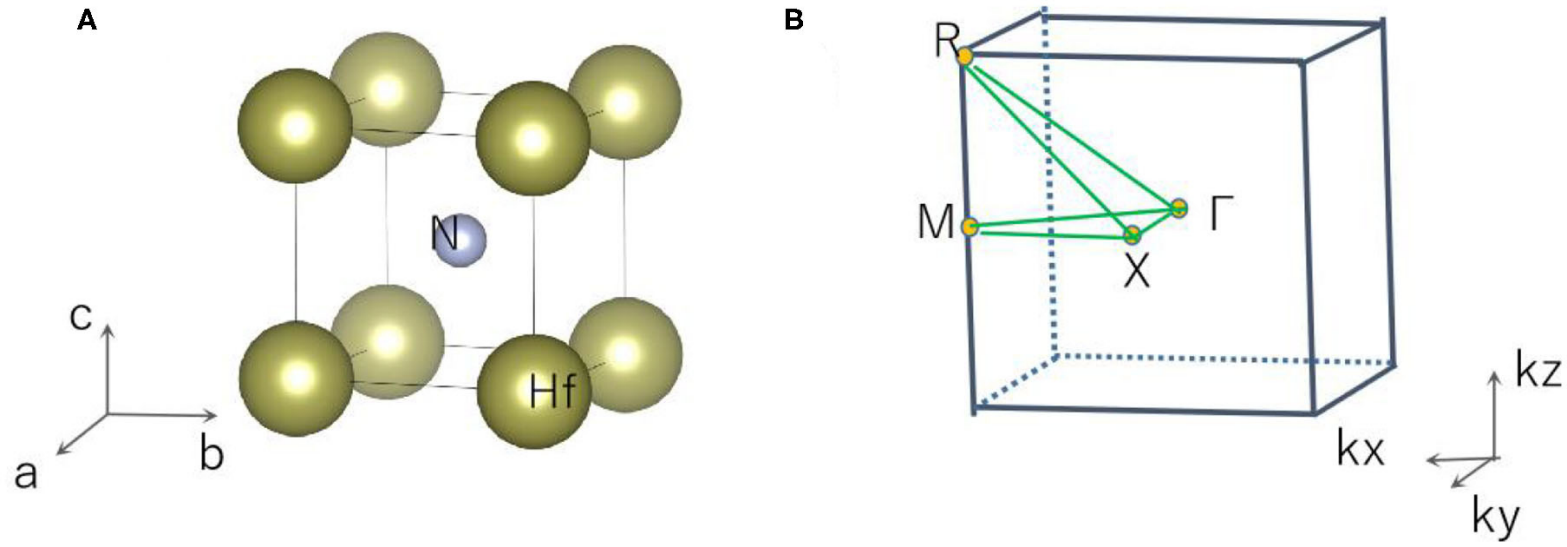
**(A)** Crystal model of HfN and **(B)** the bulk Brillouin zone.

Cubic phase HfN with space group $Pm\bar{3}m$ and ICSD: 183420^1^ was chosen for this study. The crystal structure of HfN was optimized and is shown in Figure 1A. The optimized lattice constants are a = b = c = 2.803 Å, which are in good agreement with the calculated results shown in the materials project database^1^ (a = b = c = 2.816 Å). Subsequently, we will study the electronic structure, topological elements, and surface states using the lattice constants obtained from the crystal structure.

**Figure 2:**
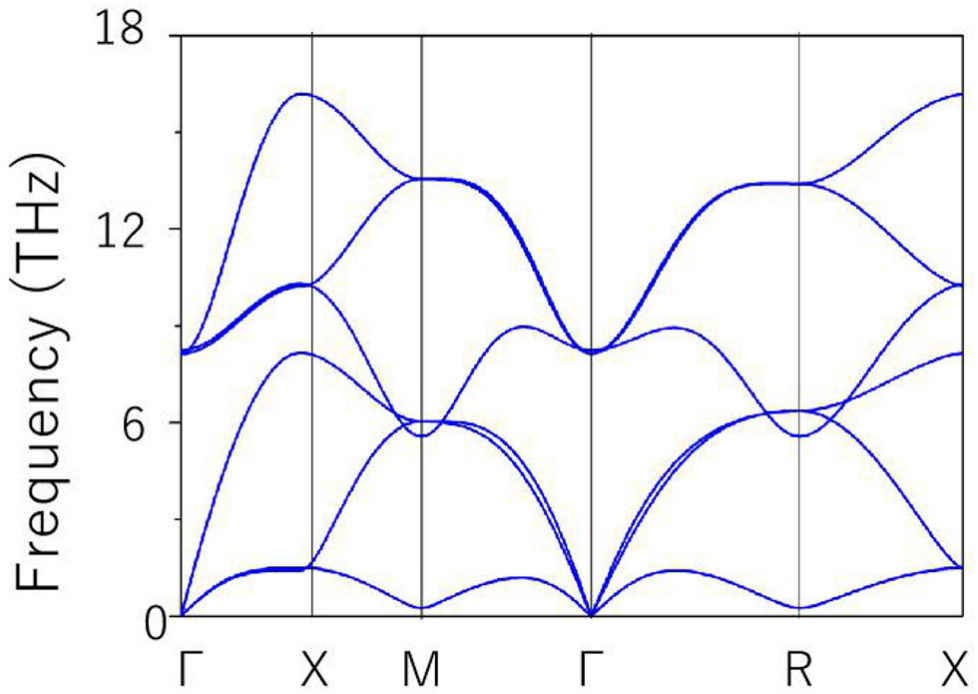
Phonon dispersion of cubic HfN material.

The 3-D bulk Brillouin zone (BZ) is shown in Figure 1B. Based on the 3-D BZ, the phonon dispersion was determined in order to examine the dynamical stability of cubic HfN material with this space group. It is noted that materials are dynamically stable when there exist no imaginary phonon modes in their phonon dispersion curves. The phonon dispersion obtained along the Γ-X-M-Γ-R-X direction is shown in Figure 2. The absence of a negative frequency in the figure confirms the dynamical stability of the HfN material.

Because HfN is a cubic system, the mechanical behavior of HfN can be achieved using three elastic constants: C_11_, C_12_, and C_44_. The results for these are as follows: C_11_ = 551.16 GPa, C_12_ = 92.96 GPa, and C_44_ = 24.31 GPa. The mechanical stability of cubic HfN can be examined based on the Born–Huang criteria as follows:

$$\begin{array}{l} \text{Criterion }1:{\text{C}}_{11}-{\text{C}}_{12}>\;0;\\ \text{Criterion }2:{\text{C}}_{11}+2{\text{C}}_{12}>\;0;\\ \text{Criterion }3:{\text{C}}_{44}>\;0. \end{array}$$

HfN is mechanically stable based on the above-mentioned Born–Huang criteria. Some other important mechanical parameters can also be obtained, such as the bulk modulus (B = 245.68 GPa), shear modulus (G = 72.03 GPa), Poisson's ratio (ν = 0.366), and Pugh's index (B/G = 3.41), based on C_11_, C_12_, and C_44_. B/G and ν are normally used to examine the brittleness and ductility of materials, with critical values of 1.75 and 0.26, respectively. For HfN, it is elastically ductile. Furthermore, the value of ν is also used to examine the nature of the chemical bonds of materials, with a critical value of 0.25; a value of ν greater (or less) than 0.25 implies an ionic (or covalent) nature. The chemical bonds of HfN are mainly ionic.

The calculated anisotropy parameter A value of 0.106 deviates from unity, reflecting the fact that HfN is elastically anisotropic. Furthermore, the directional-dependence anisotropy of Young's modulus, the shear modulus, and Poisson's ratio for HfN were obtained by means of the ELATE (Gaillac et al., 2016) program, as shown in Figures S1–S3, respectively. Young's modulus, the shear modulus, and Poisson's ratio in Figures S1–S3 all show obvious anisotropy. We would like to point out that the mechanical anisotropy of HfN is very important for its practical applications.

**Figure 3:**
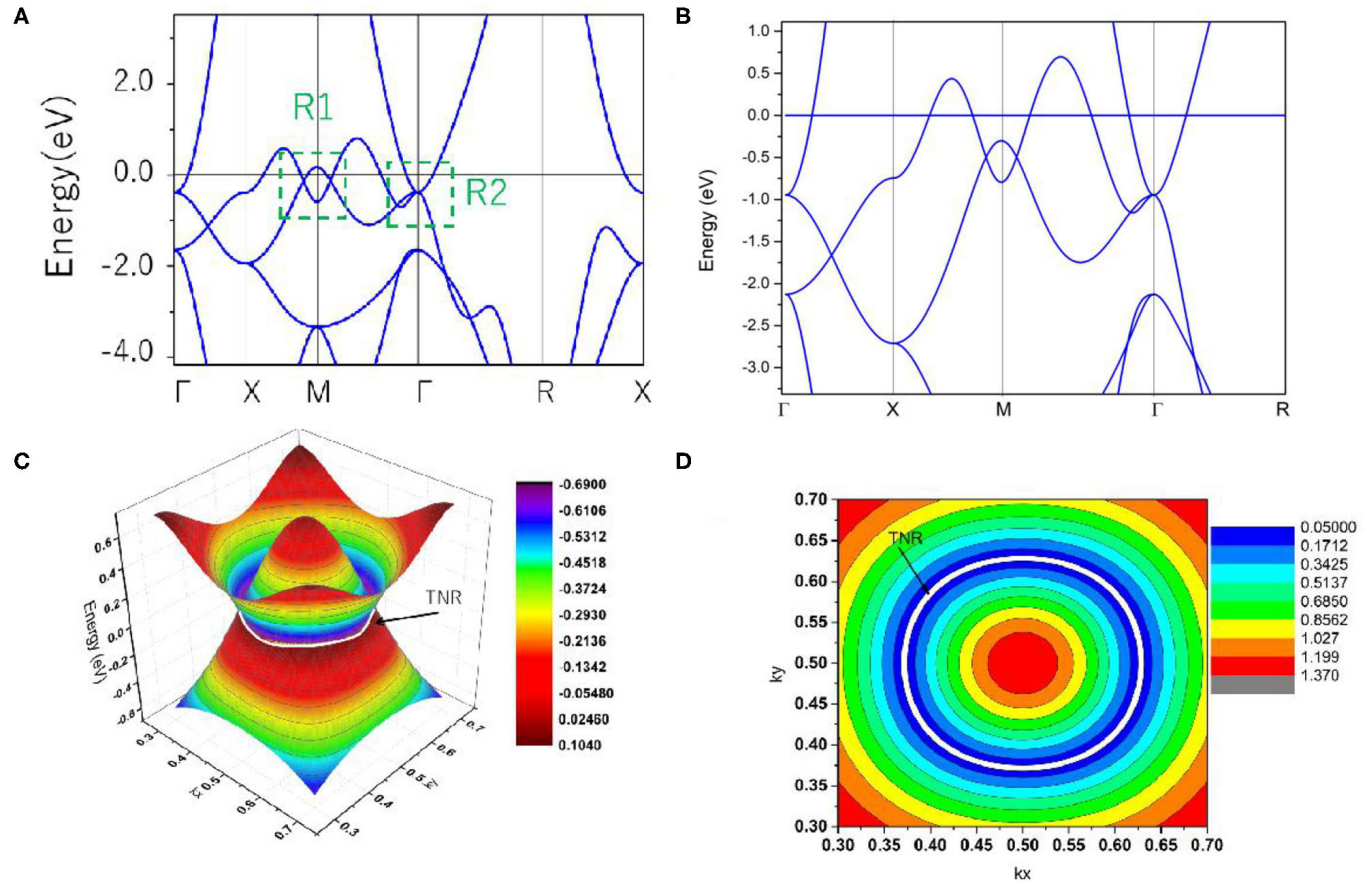
**(A)** Band structure of HfN via the GGA method; **(B)** Band structure of HfN via the HSE 06 method; **(C)** 3-D plot of both bands in the k_z_ = 0 plane in region R1; **(D)** 2-D plot of both bands in the k_z_ = 0 plane in region R1.

Based on the values of the equilibrium lattice constants obtained, we studied the electronic band structures of cubic HfN. The Γ-X-M-Γ-R-X high symmetry points (Figure 1B) in the first BZ were selected to describe the band structure of HfN. Using the generalized gradient approximation (GGA) method, the calculated band structure is shown in Figure 3A. When the spin-orbit coupling effect is ignored, we can see that HfN manifests a semimetallic band structure with conduction and valence bands overlapping each other. Interestingly, one can see that HfN possesses several band crossing points located close to the Fermi energy. We divided these band crossing points into two parts, i.e., regions R1 and R2, and we discuss these in detail separately. Before discussing the band crossing points in regions R1 and R2, the state-of-the-art Heyd–Scuseria–Ernzerhof (HSE) 06 method (Heyd and Scuseria, 2004), which can be seen as a better way of describing the band crossing points than the GGA method, was also selected to further prove the occurrence of the band crossing points in regions R1 and R2. The results of the calculated band structure of HfN with HSE 06 along the X-M-Γ-R directions are shown in Figure 3B.

First, we study the band crossing point in region R2, which indicates a band crossing point at the Γ point. This band crossing point was generated by one non-degenerate band and one doubly degenerate band. That is to say, this band crossing point is a triple nodal point, as shown in Figures 3A,B.

Next, we discuss the band crossing points along the X-M-Γ directions in region R1. Here, one can see that there are two band crossing points in total, generated by the crossing of two non-degenerate bands. Because this cubic HfN system possesses both P and T symmetries, both band crossing points in this system cannot be regarded as isolated points. Normally, both points should reside on the nodal line. To further verify that these two band crossing points in region R1 belong to a nodal line, a 3-D plot of both bands in the k_z_ = 0 plane is shown in Figure 3C. One can see that there is a closed nodal line, i.e., nodal ring, in the k_z_ = 0 plane, and the two band crossing points in region R1 belong to the closed nodal line (marked as a white ring). To confirm the shape of the closed nodal line in the kz = 0 plane, we show the 2-D plot of both bands in the k_z_ = 0 plane in Figure 3D. A near-ring shape can be seen in this figure. It is well-known that the nodal ring (Wang et al., 2020) can be classified into three types: type I, type II, and a hybrid type, according to the degree of tilting of the band crossing points in the nodal ring. Type I (or II) nodal rings are formed only by type I (or II) band crossing points, while the hybrid rings simultaneously host type I and type II band crossing points [more details about the three types of nodal ring can be found in (Wang et al., 2020)]. As shown in Figure 3C, the nodal ring in the k_z_ = 0 plane belongs to type I.

**Figure 4:**
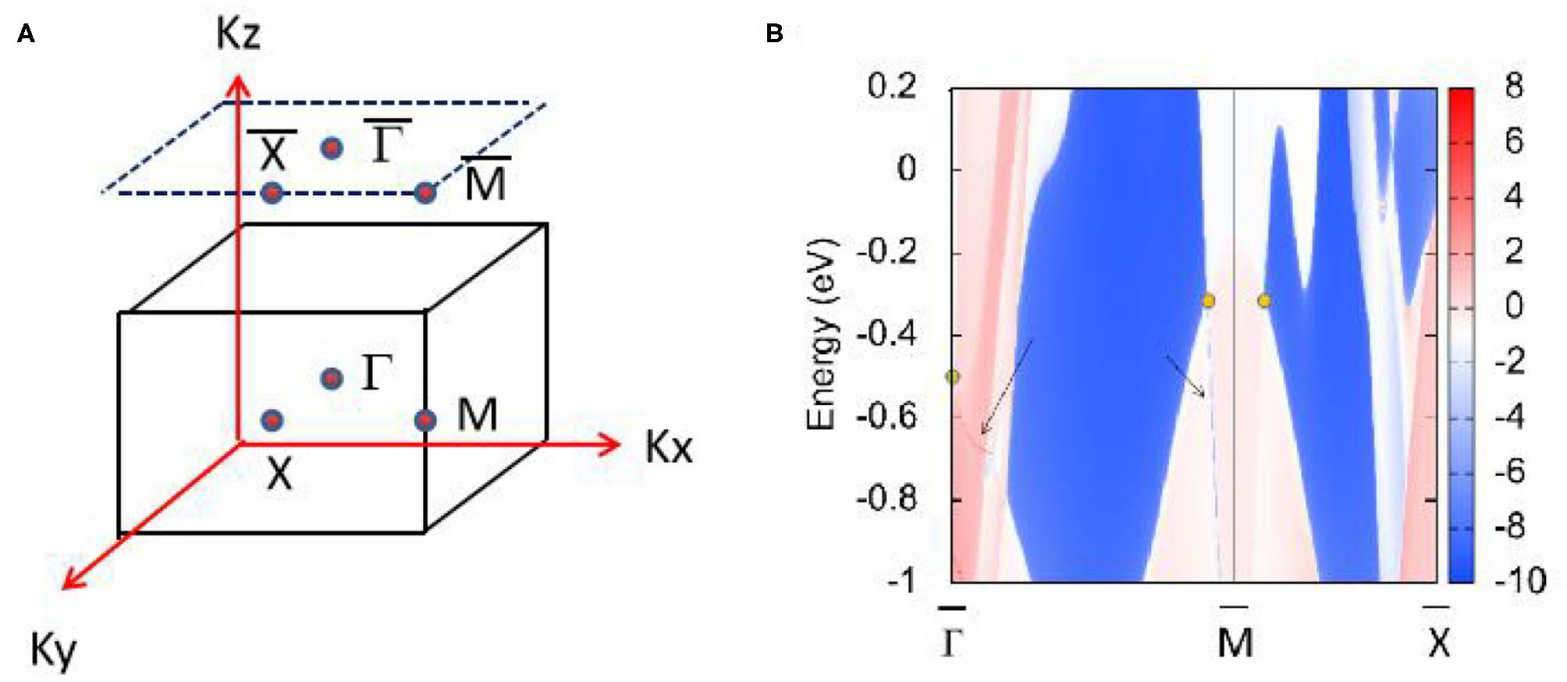
**(A)** (0 0 1) surface Brillouin zone of HfN; **(B)** Projection spectrum on the (001) surface state of HfN near the 1-D nodal ring and 0-D triple nodal point. The band crossing points are highlighted by yellow circles and the surface states are marked by black arrows.

Figure 4 shows the HfN (001) surface state without the effect of SOC, which clearly shows that topological surface states can be found around the triple nodal point at the points marked by the arrows. However, the surface state near the nodal ring is only faintly visible inside the projected topological nodal ring (see the arrow along the direction).

**Figure 5:**
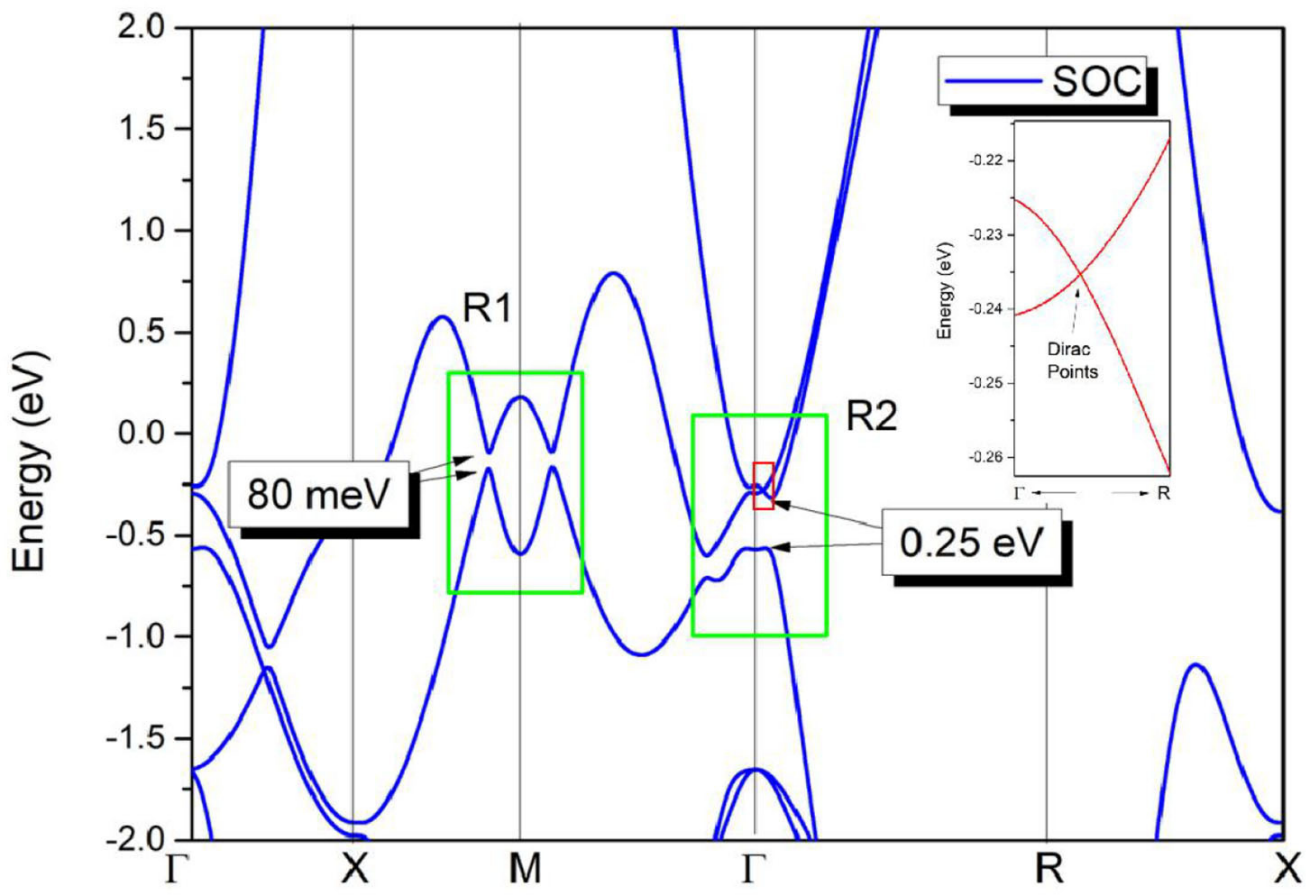
Band structure of HfN via the GGA method; The SOC effect was taken into consideration during the calculation. Insert figure: Enlarged band structure of HfN along Γ-R direction.

In the final, the effect of SOC on the electronic structure has been discussed and results are given in Figure 5. At the Γ point, an energy band gap of 0.25 eV can be found, reflecting that the triple nodal point is gapped under SOC. However, along Γ-R direction, two bands now cross with each other at−0.235 eV below the Fermi level. In this cubic HfN, the time-reversal and inversion symmetries retain, thus each band is twofold degenerate when the SOC is taken into consideration. This band-crossing point at −0.235 eV along Γ-R direction (see the insert figure in Figure 5) is a Dirac point with fourfold degeneracy. We would like to point out that similar triple nodal point - Dirac point transition can also be observed in TiB_2_ (Zhang et al., 2017) and Li_2_NaN (Jin et al., 2019b) materials.

In R1, two band crossing points are also gapped due to the SOC effect. The SOC gaps are calculated to be ~80 meV, as shown in Figure 5. We would like to point out that most nodal lines proposed so far will be gapped under the effect of SOC. The SOC-induced gaps in HfN are comparable with those in typical nodal line semimetals, such as Cu_3_NPd (Yu et al., 2015) (60–100 meV), CaAgBi (Yamakage et al., 2016) (80–140 meV), and BaSn_2_ (Huang et al., 2016) (60–160 meV).

## Conclusions

In this study, we report that cubic HfN is a newly designed topological semimetal with rich topological elements, i.e., 0-D and 1-D band crossing points. When the spin-orbit coupling (SOC) effect is ignored, near the Fermi level, HfN possesses a triple nodal point at the Γ point and a nodal ring in the k_z_ = 0 plane. The topological surface states around the 0-D and 1-D band crossing points were determined. When the SOC effect is taken into consideration, 0-D triple nodal point disappears, however, new topological signature, i.e., 0-D Dirac point occurs along Γ-R direction. The computational results also suggest that cubic HfN possesses mechanical stability, dynamical stability, elastic ductility, and anisotropy. Our investigation has revealed that HfN is a good topological semimetal with rich topological elements, high stability, and important mechanical properties.

## Data Availability Statement

All datasets generated for this study are included in the article/Supplementary Material.

## Author Contributions

YL: software, methodology, writing, and supervisor. JX: reviewing. All authors contributed to the article and approved the submitted version.

## Conflict of Interest

The authors declare that the research was conducted in the absence of any commercial or financial relationships that could be construed as a potential conflict of interest.

## Footnotes

^1^https://www.materialsproject.org/materials/mp-1001834/
