# Supplementary material for: Cubic Hafnium Nitride: A Novel Topological Semimetal Hosting a 0-Dimensional (0-D) Nodal Point and a 1-D Topological Nodal Ring
Source: Front Chem. 2020 Aug 26;8:727. doi: 10.3389/fchem.2020.00727 (PMC7479206; doi:10.3389/fchem.2020.00727)
Supplement: Supplementary file 1 [file Data_Sheet_1.docx]

# [
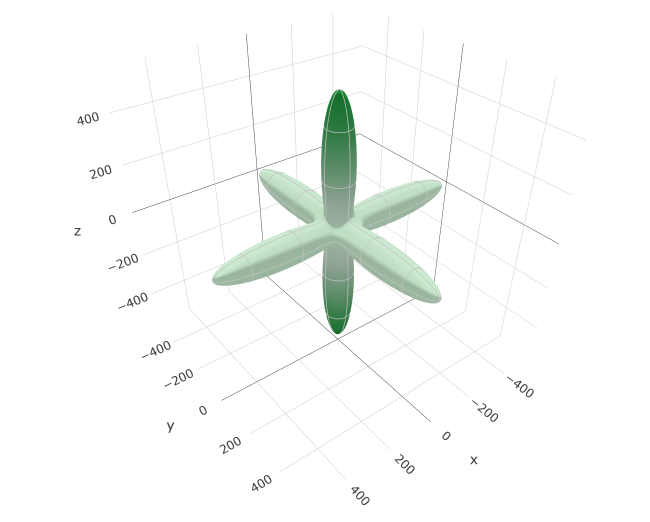
](https://pubs.rsc.org/en/content/articlelanding/2020/cp/d0cp02334e)

[
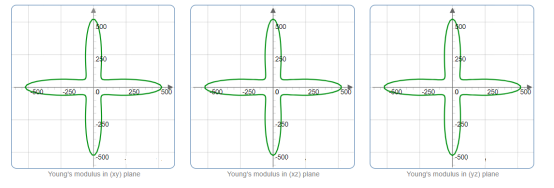
](https://pubs.rsc.org/en/content/articlelanding/2020/cp/d0cp02334e)

# [Figure S1. Directional dependence of Young’s modulus.](https://pubs.rsc.org/en/content/articlelanding/2020/cp/d0cp02334e)

# [
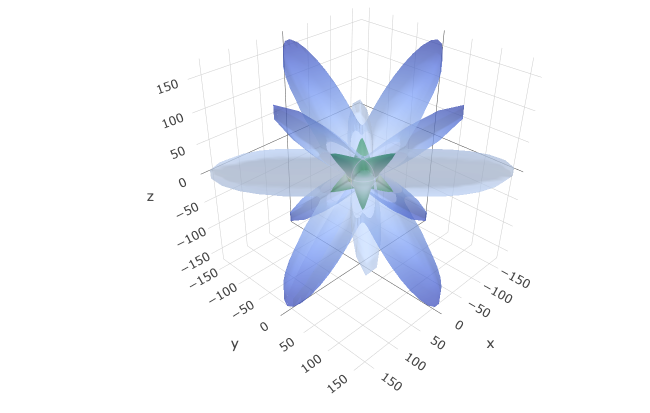
](https://pubs.rsc.org/en/content/articlelanding/2020/cp/d0cp02334e)

[
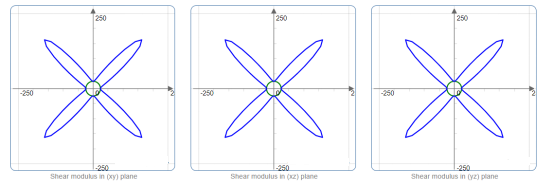
](https://pubs.rsc.org/en/content/articlelanding/2020/cp/d0cp02334e)

# [Figure S2. Directional dependence of the shear modulus: the blue and green shading show the maximum and minimum values, respectively.](https://pubs.rsc.org/en/content/articlelanding/2020/cp/d0cp02334e)

#
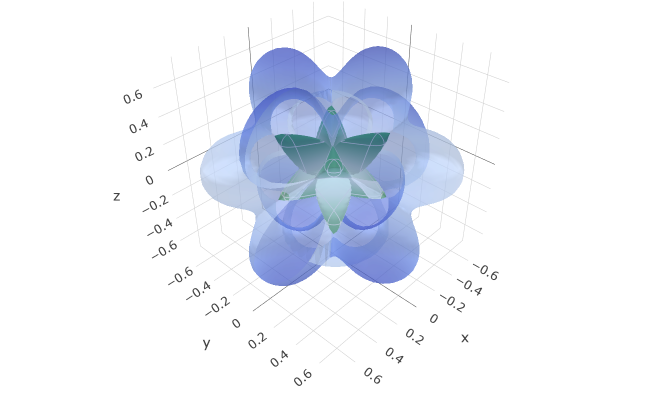


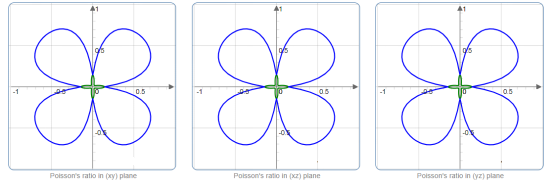


# Figure S3. Directional dependence of Poisson’s ratio: the blue and green shading show the maximum and minimum values, respectively.
